# Individual and contextual factors associated with sexual initiation among adolescents

**DOI:** 10.1186/s41155-019-0138-z

**Published:** 2019-12-23

**Authors:** Milene Fontana Furlanetto, Dienifer Mattos Ghedin, Tonantzin Ribeiro Gonçalves, Angela Helena Marin

**Affiliations:** Universidade Federal do Rio Grande do Sul, Porto Alegre, Brazil

**Keywords:** Sexuality, Sexual activity, Risk behavior, Adolescence

## Abstract

Adolescent sexual practices have been widely investigated as experiences that pose potential risks for health. The present study, therefore, aimed to investigate individual and contextual factors associated with sexual initiation, sexual activity before the age of 15 years, and inconsistent condom use. A total of 253 adolescents aged 11 to 18 years, from public schools in the capital and metropolitan region of Rio Grande do Sul, were interviewed. They answered a questionnaire covering sociodemographic data and sexual behaviors and completed the Perception of Family Support Inventory. The data were analyzed through descriptive and inferential statistics and it was verified that both individual (higher level of education, school repetition, and use of licit and illicit substances) and contextual factors (perception of less affective-consistent support and greater autonomy from the family) were related to sexual initiation among adolescents. The associations found for the groups with sexual initiation before and after 15 years of age were similar, indicating that age did not increase the exposure to risks. Inconsistent condom use was related to the use of tobacco and other drugs. Taken together, the results indicated the co-occurrence of risk behaviors, such as sexual risks and substance abuse, as well as highlighting some family characteristics as protective factors.

## Introduction

Among the stages of the life cycle, adolescence has been associated with concerns about harm prevention and health promotion. Adolescents have been considered a vulnerable group for the acquisition of sexually transmitted infections (STIs) and unplanned pregnancies, mainly because they are seen as immature for making decisions related to their health (Spencer, Doull, & Shoveller, 2012). Accordingly, one of the main intervention strategies is the transmission of preventive information, aiming to promote healthy sexual behaviors. However, studies have indicated the lack of effectiveness of some of these actions, especially since they do not consider the social, familial, and affective contexts of the adolescents (Moura et al., 2013; Spencer et al., 2012).

In 2014, the World Health Organization (WHO) highlighted the need for countries to invest in health strategies for this population, as the main cause of death among adolescents was related to HIV infection/AIDS and other risk behaviors, such as motor vehicle accidents and suicidal behavior (WHO, 2014). In Brazil, an increasing trend for HIV infection/AIDS among boys aged 15 to 19 was found. The number of cases increased from 2.4 to 6.7 cases per 100,000 inhabitants in the previous 10 years (Brasil, 2017). Similarly, in the last national Survey of Knowledge, Attitudes and Practices (Pesquisa de Conhecimentos, Atitudes e Práticas—PCAP) (Brasil, 2013) respondents aged 15 to 24 presented a mean of 73.1% correct answers regarding forms of HIV transmission and prevention. However, only 61.0% had used condoms during their first sexual intercourse and only 35.1% used them with a steady partner, with 58.8% using them in the last sexual relationship with a casual partner (Brasil, 2013). Comparing the data from the 2013 survey with those from 2004, there was a decrease in condom use according to the age of the participant and the type of intimate relationship, with stable relationships being associated with a higher risk for inconsistent use (Brasil, 2013). These data highlight the need to better understand the risk factors involved in sexual relations among adolescents.

The main sexual risk behaviors investigated in the literature have been the inconsistent condom use and early sexual initiation (e.g., before the age of 15) (Dallo & Martins, 2018; Espada, Morales, & Orgilés, 2014). Not using condoms exposes adolescents to STIs or unplanned pregnancies. Younger age at sexual initiation is associated with emotional immaturity and a lower perception of risk and of the consequences of the actions, making these adolescents more vulnerable to group influences (Espada et al., 2014; González, Montero, Martínez, Mena, & Varas, 2010; Siegel, Lekas, de Ramjohn, Schrimshaw, & VanDevanter, 2014).

It is important to consider that risk situations involve factors other than access to information and characteristics typical of adolescence. For example, in contexts of greater social vulnerability, access to health services and education, which tends to be more precarious, leads to different opportunities to risk exposure when compared to more favorable socioeconomic contexts (WHO, 2014). For example, studies with adolescents living in the street or in institutions indicate a greater precocity of sexual relations when compared to adolescents living with their families (Zappe, Alves, & Dell’Aglio, 2018).

Sexual risk-taking may also be related to the family context. Adequate parental supervision, understood as not exceeding extremes of control or neglect, allied with positive and open dialog between the parents and children are related to the adolescent’s health self-care behaviors (Wang et al., 2013). However, when adolescents face inflexible patterns when seeking autonomy, lack of emotional support, and communicational difficulties with their parents, risk-taking sexual activity may be greater (Siegel et al., 2014; Wang et al., 2013).

Family support is defined as a set of factors that include the encouragement of autonomy, affection, cooperation, and the organization of family rules. Family support was associated with risk behaviors and the mental health of university students in the study by Souza, Baptista, and Baptista (2010). Perception of low family support was significantly associated with higher reports of common mental disorders, higher frequency of risky sexual behaviors, self-harm, and lack of leisure activities (Souza et al., 2010).

In addition, studies have highlighted that health risk behaviors among adolescents may occur in more than one area, suggesting that there may be a causal sequence between them (Zappe et al., 2018). For example, increased sexual risk has been consistently associated with the use of licit and illicit substances, such as tobacco, alcohol, marijuana, and other drugs (Zappe & Dell’Aglio, 2016). Drug use has been related to early sexual initiation (González et al., 2010; Ruiz, Molinero, Miguelsanz, & Rodriguez, 2015) and to inconsistent condom use (Oliveira-Campos et al., 2014), together with greater social vulnerability and perceptions of more negative family relationships (Zappe & Dell’Aglio, 2016), lack of school attendance and dropout, and delinquent and offending behavior (Zappe et al., 2018).

Adherence to preventive measures does not only depend on individual characteristics and the personal choices of young people but it is also associated with a number of contextual factors. Although studies show that the diffusion of preventive information increases knowledge about condom use, educative interventions do not occur consistently and tend to vary according to social strata (Brasil, 2013). Accordingly, the aim was to contemplate individual and contextual variables related to these sexual behaviors, especially the family, since most preventive programs only focus on individual behavior (Furlanetto, Lauermann, da Costa, & Marin, 2018; Spencer et al., 2012). It is expected that the data can help identify risk and protective factors to be included in adolescent health promotion strategies from a systemic perspective.

Therefore, the objective of the present study was to investigate the association between sexual activity in adolescence and individual factors (gender, age, and education, history of school repetition, religion, and use of licit and illicit drugs) and contextual factors (family configuration, family support, socioeconomic status of the family, parental education, and access to information about sex and sexuality). In addition, among the adolescents who had already initiated sexual activity, the association of individual and contextual factors with early sexual initiation and inconsistent condom use was examined.

## Method

### Design and participants

This was an observational, cross-sectional study (Grimes & Shulz, 2002) with a quantitative approach. A convenience sample was selected from four state elementary schools in the city of Porto Alegre, capital of the state of Rio Grande do Sul, and two municipal schools in the city of São Leopoldo, also in Rio Grande do Sul, following the indication of the local Department of Education and their geographical location. As inclusion criteria, the adolescents had to be enrolled in years 6 through 9 of elementary education and could not be over 18 years of age. Approximately 1000 students were approached and invited to participate in the study. The contemplated schools were accessed for convenience.

The adolescents were included in the study after obtaining the permission of their parents/guardians. The students took home a consent form that contained data regarding the research topics and collection procedures, and 256 authorizations were returned after being signed. The large sample loss occurred mainly because the parents or adolescents did not feel comfortable with the subject of sexuality, with this issue still appearing to be taboo in most families. Furthermore, to answer the questionnaire, the participant had to have cognitive ability compatible with their peers. To avoid ethical concerns, all students in the room were invited to participate and, subsequently, three questionnaires of adolescents with special needs, such as mental retardation, were excluded. This diagnosis was communicated by the school or by the parents themselves.

Therefore, 253 adolescents (61% girls and 39% boys) from both cities participated, presenting a mean age of 13.67 years (SD = 1.54). The majority of the adolescents (52.8%) had a higher middle-class socioeconomic status (B1 and B2), followed by lower middle class (37.7%, C1 and C2), according to the *Critério Brasil.*^1^ There were no significant differences (Mann-Whitney *U* test) between girls and boys in relation to the independent variables. The number of participants was estimated to comprise 15 to 20 subjects for each independent variable included in the regression analyses, as proposed by Hair, Black, Babin, Anderson, and Tatham (2009).

### Data collection instruments and procedures

The participants were approached in the classroom and asked to answer a questionnaire on sociodemographic data and sexual behavior, developed by the authors. The questionnaire investigated socioeconomic aspects, knowledge about sexuality, sexual behavior, and information and access to sexual education programs, as well as communication about sexuality in the family and at school. The appropriateness and clarity of the questions were evaluated through a pilot study with approximately 10% of the total number of participants. The questionnaire was explained before the application and participants could express doubts during completion.

The adolescents also completed the Perception of Family Support Inventory (*Inventário de Percepção e Suporte Familiar*–IPSF; Baptista, 2009), a self-applied measure developed in Brazil, composed of 42 items assessing three dimensions of the family support. The affective-consistent dimension contains 21 items and addresses the positive relationships within the family, including the interest for the others, verbal and nonverbal expressions of affection, clarity of roles, respect for family rules, and ability to cope with problematic situations (α = 0.91). The family adaptation dimension (13 items) includes negative perceptions about family feelings and behaviors, such as anger, isolation, aggression, shame, competition, and guilt among the members (α = 0.90). This dimension is scored inversely, that is, the higher the score, the lower the feelings of isolation, aggressive relationships, and misunderstanding. Finally, the family autonomy dimension has eight items that evaluate autonomy perception in relation to the family, denoting relationships of trust, privacy, and freedom among the members (α = 0.78). The inventory is answered on a 3-point Likert scale, scoring from 0 to 84 points. Considering the participant’s gender, the score obtained is also classified in low, medium-low, medium-high, and high levels for each dimension (Baptista, 2009). Both instruments were applied in random order and completed individually, taking from 30 to 50 min.

### Ethical procedures

The study was approved by the Research Ethics Committee of Vale do Rio dos Sinos University (protocol 66,618,717.9.0000.5344). All ethical considerations required for studies with human subjects were followed, according to Resolution 510/16 of the National Health Council (Brasil, 2016).

The Municipal Department of Education of São Leopoldo and the State Department of Education of the Rio Grande do Sul approved the study. Both indicated three of the schools to be included in the study and others were selected due to the ease of access. After acceptance, the project was presented to the pedagogical schools’ teams, who suggested the best times to approach the students. The students only participated in the research after the parent or guardian had signed and returned the informed consent form. Adolescents also signed an assent form, which includes the study objectives, procedures, and other explanations regarding confidentiality. Both forms informed they would be assisted for free by the psychology team of the Outpatient Health Care Project (UNISINOS) in case any difficulty or problem arose due to the participation in the study. Also, the participants were informed that they could refuse to participate or withdraw their consent at any time without any prejudice. However, there was no need or request for any referral.

### Data analysis procedures

The main outcome variable of the study was sexual initiation, which was measured from the question “have you ever had a sexual intercourse?”. Among the group of adolescents who reported having had sex (*n* = 80), two additional outcomes variables were investigated: early sexual initiation, before the age of 15 (*n* = 54), and inconsistent condom use, defined as no condom use either at the first sexual intercourse and/or at the last intercourse (*n* = 32). As independent variables, individual factors (gender, age, education, history of school repetition, religion, and use of licit and illicit drugs) and contextual factors (family configuration, family support, family socioeconomic level, parental education, and access to information about sex and sexuality) were investigated. The data were analyzed using the Statistical Package for the Social Sciences, version 20.0, program for Windows, adopting a 5% level of significance for statistical decision criteria (SPSS Inc, n.d.).

Initially, the absolute (*n*) and relative (%) frequencies, as well as the measures of central tendency and dispersion (mean and standard deviation) were obtained for all variables. Bivariate analyses were performed using Pearson’s chi-square test (ϰ^2^) or Fisher’s exact test, as well as the Mann-Whitney *U* test. Subsequently, Multivariate Binary Logistic Regression analysis was performed for the main outcome variable and all variables with *p* < 0.20 in the bivariate analyses were included in the model. The conditional backward method was used, where the odds ratio and *p* value estimates were adjusted for the variables that composed each step of the models generated. The goodness of fit of the final model was evaluated from the *R*^*2*^ values of Nagelkerke and Hosmer-Lemeshow. The probability of gradual entry of the variables into the model was 0.05 and for their removal 0.10. Regarding the cutoff point, the significance was 0.50 for the maximum of 20 interactions. Levels of significance lower than 0.01 were considered significant based on the Bonferroni criterion. The estimate of the effect measure was expressed in the crude odds ratios (OR) and the 95% confidence interval (95% CI). It was not possible to test regression models with secondary outcomes (early sexual intercourse and inconsistent condom use) because of the low number of participants who reported these situations among those who had had sex.

## Results

The results are presented in two parts. Part I presents the analyses of the association between sexual initiation in adolescence and individual and contextual factors. Part II prioritized only those adolescents that had already had sexual relations and presents the analysis of association between individual and contextual factors and, respectively, early sexual initiation and inconsistent condom use.

### Part I: Sexual initiation and associated individual and contextual factors

The participation according to the school year was well distributed, with the 7th grade being the one with most participants (29.6%). Most of the adolescents had never repeated a year at school (61.3%), were religious, but did not consider themselves practicing (49.0%).

Alcohol use was reported by 43.1% of the adolescents and only 10.3% of them reported the use of tobacco and illicit drugs at least once in their lifetime (Table 1). About one-third of the participants (31.6%; 44 girls and 36 boys) had already had sexual relations. The mean age of sexual debut was 13.60 years (SD = 1.33), 13.70 years (SD = 1.3) for girls, and 13.45 (SD = 1.2) for boys. The frequency of sexual intercourse was once or less per month (72.8%). Vaginal sex was the most mentioned sexual practice (85.9%), followed by oral sex (59.0%), mutual masturbation (21.8%), anal sex (20.5%), and sex with more than one person (5.1%).

**Table 1:** Comparisons of the individual factors between adolescents with and without sexual initiation (*N* = 253)

Individual factors		Sexual initiation		
	Total % (*n*)	Yes % (*n*)	No % (*n*)	*p*
Gender				
Boys	39.1 (99)	36.4 (36)	63.6 (63)	0.19
Girls	60.9 (154)	28.6 (44)	71.4 (110)	
Schooling				
6th year	14.6 (37)	16.2 (6)	*83.8 (31)*	0.01*
7th year	29.6 (75)	26.7 (20)	73.3 (55)	
8th year	27.3 (69)	30.4 (21)	69.6 (48)	
9th year	28.5 (72)	*45.8 (33)*	54.2 (39)	
School repetition				
Never repeated	61.3 (155)	16.1 (25)	*83.9 (130)*	0.00*
Repeated 1 time	22.5 (57)	*45.6 (26)*	54.4 (31)	
Repeated 2 times	11.1 (28)	*64.3 (18)*	35.7 (10)	
Repeated 3 times or more	5.1 (13)	*84.6 (11)*	15.4 (2)	
Religion				
Yes, practicing	30.8 (78)	29.5 (23)	70.5 (55)	0.88
Yes, not practicing	49.0 (124)	32.3 (40)	67.7 (84)	
No	20.2 (51)	33.3 (17)	66.7 (34)	
Alcoholic beverages				
No	56.6 (142)	15.5 (22)	*84.5 (120)*	0.00*
Yes	43.1 (109)	*52.3 (57)*	47.7 (52)	
Tobacco				
No	89.7 (226)	28.3 (64)	*71.7 (162)*	0.00*
Yes	10.3 (26)	*61.5 (16)*	38.5 (10)	
Illicit drugs				
No	89.7 (227)	27.8 (63)	*72.2 (164)*	0.00*
Yes	10.3 (26)	*65.4 (17)*	36.6 (9)	

*Significant data. Chi-square test

The percentage of students who had already had sexual intercourse increased according to schooling, with the majority concentrated in the 9th grade (45.8%; *n* = 33) [ϰ^2^ (3) = 11.68; *p* = 0.009]. To have repeated a year at school, whether one (22.5%; *n* = 57), two (11.1%; *n* = 28) or three times (5.1%; *n* = 13), was significantly associated with the initiation of sexual activity [ϰ^2^ (3) = 53.07; *p* = 0.00], as well as the use of alcohol [ϰ^2^ (1) = 38.72; *p* = 0.00], tobacco [ϰ^2^ (1) = 11.07; *p* = 0.00] and other drugs [ϰ^2^ (1) = 15.27; *p* = 0.00]. Table 1 presents the absolute, relative, and comparative frequencies of the variables associated with sexual initiation.

Considering contextual factors, the adolescents’ socioeconomic level was concentrated in the middle stratum (B2 = 31.1%, C1 = 27%, and B1 = 21.7%) and the parents’ education level was, for the most part, equal to incomplete and complete high school education (Table 2). The nuclear family configuration, consisting of a father, mother, and children, represented 38.5% of the sample, followed by the extended family configuration, formed by other relatives as well as the parents, with 22.2%. Single-parent families (e.g., mother and children or the father and children) and remarried families represented approximately 20.0% each.

**Table 2:** Comparisons of the contextual factors between adolescents with and without sexual initiation (*n* = 253)

Contextual factors	Sexual initiation			
	Total % (*n*)	Yes % (*n*)	No % (*n*)	*p*
Socioeconomic level				
A	7.0 (17)	41.2 (7)	58.8 (10)	0.35
B1	21.7 (53)	22.6 (12)	77.4 (41)	
B2	31.1 (76)	31.6 (24)	68.4 (52)	
C1	27.0 (66)	39.4 (26)	60.6 (40)	
C2	10.7 (26)	26.9 (7)	73.1 (19)	
D	2.5 (6)	50 (3)	50 (3)	
Mother’s education				
Incomplete elementary	16.7 (42)	48.7 (20)	53.6 (22)	0.13
Complete elementary	12.2 (30)	36.7 (11)	63.3 (19)	
Incomplete high school	9.0 (22)	36.4 (8)	63.6 (14)	
Complete high school	20.0 (49)	26.5 (13)	73.5 (36)	
Incomplete higher education	9.0 (22)	31.8 (7)	68.2 (15)	
Complete higher education	32.7 (80)	23.8 (19)	76.3 (61)	
Father’s education				
Incomplete elementary	19.3 (47)	38.2 (18)	61.7 (29)	0.17
Complete elementary	12.4 (29)	37.9 (11)	62.1 (18)	
Incomplete high school	4.3 (10)	50 (5)	50 (5)	
Complete high school	23.6 (55)	21.8 (12)	78.2 (43)	
Incomplete higher education	8.6 (20)	40 (8)	60 (12)	
Complete higher education	30.9 (72)	25 (18)	75 (54)	
Family configuration				
Nuclear	38.5 (97)	19.6 (19)	*80.4 (78)*	0.01*
Extended	22.2 (56)	*41.1 (23)*	58.9 (33)	
Single parent	20.2 (51)	39.2 (20)	60.8 (31)	
Remarried	19 (48)	37.5 (18)	62.5 (30)	
Affective-consistent				
Low	26.3 (66)	39.4 (26)	60.6 (40)	0.15***
Medium low	21.1 (53)	28.3 (15)	71.7 (38)	
Medium high	29.5 (74)	29.7 (22)	70.3 (52)	
High	23.1 (58)	29.3 (17)	70.7 (41)	
Adaptation				
Low	30.3 (76)	39.5 (30)	60.5 (46)	0.07***
Medium low	27.9 (70)	35.7 (25)	64.3 (45)	
Medium high	23.5 (59)	28.8 (17)	71.2 (42)	
High	18.3 (46)	17.4 (8)	82.6 (38)	
Autonomy				
Low	42.2 (106)	22.6 (24)	*74.7 (82)*	0.00***
Medium low	36.7 (92)	32.6 (30)	67.4 (62)	
Medium high	17.1 (43)	*41.9 (18)*	58.1 (25)	
High	4 (10)	*80 (8)*	20 (2)	
Total family support				
Low	36.7 (92)	33.7 (31)	66.3 (61)	0.45
Medium low	24.7 (62)	30.6 (19)	69.4 (43)	
Medium high	24.7 (62)	33.9 (21)	66.1 (41)	
High	13.9 (35)	25.7 (9)	74.3 (26)	
Access to information about sexuality in school				
No	24.5 (62)	33.8 (21)	66.2 (41)	0.66
Yes	75.5 (191)	30.8 (59)	69.2 (132)	
Access to information about sexuality in the family				
No	24.5 (62)	20.9 (13)	79.1 (49)	0.09
Yes	75.5 (191)	35.0 (67)	65.0 (123)	

*Significant data. Chi-square test

** Significant data. Mann-Whitney

It was found that most of the adolescents perceived their total family support as low (36.7%). However, it should be highlighted that the sample was composed of children below 18 years of age and, therefore, the autonomy dimension was expected to show low results, which was reflected in the total score. Thus, the dimensions were considered individually in the association analyses. Finally, the majority of the participants reported that they had received information about sex and sexuality from the family and at school.

A significant relationship between sexual initiation and the extended family configuration was found, whereas non-initiation was associated with the nuclear family [ϰ^2^ (3) = 10.91; *p* = 0.012]. There was also an association between having had sexual intercourse and medium-high and high perception of family autonomy [ϰ^2^ (3) = 16.82; *p* = 0.001]. Table 2 presents the absolute and relative frequencies and the comparison of the contextual variables related to sexual initiation.

The predictive role of individual (gender, education, school repetition, use of alcoholic beverages, tobacco, and/or drugs) and contextual factors (mother’s education, father’s education, family configuration, family support dimensions, and access to information about sex and sexuality) in relation to sexual initiation were analyzed through Multivariate Binary Logistic Regression Analysis. All variables with a minimum level of significance of *p* ≤ 0.20 were included in the initial model.

The possible effect of distinct profile characteristics of the sample on the study outcome considering the different schools included was tested. Accordingly, the final model was adjusted for the confounding variables detected, which were gender, school grade of the student, and socioeconomic class. For these variables, the difference between the estimates of gross and adjusted odds ratios was higher than 10%. Among the individual factors, the results of the adjusted final model, presented in Table 3, show that having one or two school repetitions significantly explained sexual initiation, as well as alcohol use, estimated a risk more than three times greater.

**Table 3:** Logistic regression for associations between sexual initiation and individual and contextual factors (*n* = 253)

Final model	Regression coefficient			OR_Adjusted_		
	B_crude_	S.E	Sig.	OR	95% CI	
					Below	Above
Use of alcoholic beverages	1.39	0.51	*0.00*	3.81	1.77	8.2
School repetition						
Once	1.60	0.58	*0.00*	4.46	1.87	9.4
Twice	2.17	0.81	*0.00*	6.89	2.05	17.34
Family configuration						
Single parent	1.35	0.66	*0.01*	3.67	1.36	9.90
Extended	2.17	0.64	*0.00*	6.85	2.61	17.97
Remarried	1.01	0.70	0.05	2.03	1.00	6.46
Family support						
Affective-consistent	−0.08	0.03	*0.01*	0.85	0.70	0.95
Autonomy	0.32	0.09	*0.00*	1.68	1.45	1.93
Constant	10.91	3.16	0.00			

School year and *Critério Brasil* (confounding factors); initial model—note: Nagelkerke’s R^2^ 0.529; Hosmer-Lemeshow test (chi-square = 8.007; *p* = 0.433); Cox & Snell: 0.344; general proportion of correct responses—confusion matrix: 75.2%; final model—note: Nagelkerke’s R2 0.570; Hosmer-Lemeshow test (chi-square = 12.904; *p* = 0.115); Cox & Snell: 0.456; general proportion of correct responses-confusion matrix: 86.1%

*Regression models adjusted for the school

Among the contextual factors, the single-parent and extended family configurations remained representative in the final model, indicating a probability three and almost seven times higher, respectively, of sexual initiation among these adolescents. Higher scores in the affective-consistent dimension of family support decreased the chance of sexual initiation by 15%, whereas higher autonomy scores increased this probability 1.6 times.

The final regression model presented good indicators. The Cox and Snell tests indicated that this explained 45.6% of the variances in the sexual initiation, whereas Nagelkerke’s estimation indicated that the model explained 57% of the observed variations. The Hosmer-Lemeshow test did not show significant differences between the model estimates and the actual classifications of the sample [ϰ^2^ (10) = 12.90; *p* = 0.11]. Finally, in the confusion matrix, the total number of correct answers was 84.5%, considering that the final model correctly classified 74.8% of the cases that had already had sex and 94.3% of the cases in which the answer was negative.

### Part II: Early sexual initiation, inconsistent condom use, and associated individual and contextual factors

A total of 54 adolescents (29 girls and 25 boys) reported that their sexual debut occurred before the age of 15, representing 67.5% of the sample that had already had intercourse (*n* = 80). For individual factors, findings were similar to those encountered for sexual initiation outcome (Part I), since sexual precocity was associated with grade repetition [ϰ^2^ (9) = 72.19; *p* < 0.001] and the use of alcohol [ϰ^2^ (3) = 38.32; *p* < 0.001], tobacco [ϰ^2^ (3) = 13.28; *p* = 0.002], and/or other drugs [ϰ^2^ (3) = 17.48; *p* < 0.001].

Inconsistent condom use was reported by 40% of the adolescents (17 girls and 15 boys), with 74.7% having used condoms during the first sexual intercourse and 67.1% in the last one. There was a positive association between inconsistent condom use and the use of tobacco [ϰ^2^ (1) = 5.05; *p* = 0.02] and other drugs [ϰ^2^ (1) = 3.84; *p* = 0.05].

Among the contextual factors, it was evidenced that early sexual initiation was associated with an extended family configuration [ϰ^2^ (6) = 12.75; *p* = 0.04] and with higher scores in the autonomy dimension of the family support [ϰ^2^ (6) = 24.42; *p* < 0.001] (Tables 4 and 5).

**Table 4:** Comparisons of the individual factors considering early sexual initiation and inconsistent use of condoms (*n* = 80)

Individual factors	Early sexual initiation			Inconsistent use		
	Yes (*n* = 54)	No (*n* = 26)	*p*	Yes (*n* = 32)	No (*n* = 48)	*p*
	% (*n*)	% (*n*)		% (*n*)	% (*n*)	
Gender			0.36			0.91
Boys	86.2 (25)	13.7 (4)		41.7 (15)	58.3 (21)	
Girls	74.3 (29)	25.6 (10)		40.5 (17)	59.5 (25)	
Schooling			0.5			
6th year	100 (6)	0.0 (0)		33.3 (2)	66.7 (4)	0.96
7th year	83.3 (15)	16.6 (3)		40.0 (8)	60.0 (12)	
8th year	82.3 (14)	17.6 (3)		45.0 (9)	55.0 (11)	
9th year	74.1 (20)	25.9 (7)		40.6 (13)	59.4 (19)	
School repetition						
Never repeated	100 (23)	*0.0 (0)*	0.00**	29.2 (7)	70.8 (17)	0.09
Repeated 1 time	88.5 (23)	*11.5 (3)*		52.0 (13)	48.0 (12)	
Repeated 2 times	*68.8 (11)*	31.1 (5)		55.6 (10)	44.4 (8)	
Repeated 3 times or more	*44.4 (4)*	55.6 (5)		18.2 (2)	81.8 (9)	
Religion						
Yes. practicing	90.4 (19)	9.5 (2)	0.66	47.8 (11)	52.2 (12)	0.58
Yes. Not practicing	74.2 (26)	25.7 (9)		41.0 (16)	59.0 (23)	
No	75.0 (9)	25.0 (3)		31.3 (5)	68.8 (11)	
Alcoholic beverages						
No	78.9 (15)	21.1 (4)	0.00**	33.3 (7)	66.7 (14)	0.37
Yes	*79.5 (39)*	*20.4 (10)*		44.6 (25)	55.4 (31)	
Tobacco						
No	81.1 (43)	18.8 (10)	0.00**	34.9 (22)	*65.1 (41)*	0.02*
Yes	*73.3 (11)*	*26.6 (4)*		*66.7 (10)*	33.3 (5)	
Illicit drugs						
No	83.0 (44)	16.9 (9)	0.00**	35.5 (22)	*64.5 (40)*	0.05*
Yes	*66.6 (10)*	*33.3 (5)*		*62.5 (10)*	37.5 (6)	

*Chi-square test

**Fischer’s exact test

**Table 5:** Comparisons of the contextual factors considering early sexual initiation and inconsistent use of condoms (*n* = 80)

Contextual factors	Early sexual initiation			Inconsistent use		
	Yes (*n* = 54)	No (*n* = 26)	*p*	Yes (*n* = 32)	No (*n* = 48)	*p*
Socioeconomic level			0.7			0.37
A	71.4 (5)	28.5 (2)		50.0 (3)	50.0 (3)	
B1	83.3 (10)	16.6 (2)		33.3 (4)	66.7 (8)	
B2	66.6 (19)	33.3 (3)		26.1 (6)	73.9 (17)	
C1	73.6 (14)	26.3 (5)		53.8 (14)	46.2 (12)	
C2	85.7 (6)	14.28 (1)		57.1 (4)	42.9 (3)	
D	66.6 (2)	33.3 (1)		33.3 (1)	66.7 (2)	
Mother’s education			0.5			0.18
Incomplete elementary	68.7 (11)	31.2 (5)		30.0 (6)	70.0 (14)	
Complete elementary	85.7 (6)	14.2 (1)		36.4 (4)	63.6 (7)	
Incomplete high school	85.7 (6)	14.2 (1)		62.5 (5)	37.5 (3)	
Complete high school	83.3 (10)	16.6 (2)		53.8 (7)	46.2 (6)	
Incomplete higher education	83.3 (5)	16.6 (1)		71.4 (5)	28.6 (2)	
Complete higher education	77.7 (14)	22.2(4)		23.5 (4)	76.5 (13)	
Father’s education			0.31			0.65
Incomplete elementary	86.6 (13)	13.3 (2)		50.0 (9)	50.0 (9)	
Complete elementary	70.0 (7)	30.0 (3)		27.3 (3)	72.7 (8)	
Incomplete high school	100 (4)	0.0 (0)		40.0 (2)	60.0 (3)	
Complete high school	72.7 (8)	27.3 (3)		54.5 (6)	45.5 (5)	
Incomplete higher education	66.6 (4)	33.3 (2)		25.0 (2)	75.0 (6)	
Complete higher education	76.4 (13)	23.5 (4)		47.1 (8)	52.9 (9)	
Family configuration			0.04**			0.98
Nuclear	87.5 (16)	12.5 (2)		44.4 (8)	55.6 (10)	
Extended	*85.7 (18)*	14.2 (3)		40.9 (9)	59.1 (13)	
Single parent	*66.6 (12)*	*33.3 (6)*		40.0 (8)	60.0 (12)	
Remarried	76.9 (10)	23.0 (3)		38.9 (7)	61.1 (11)	
Affective-consistent			0.93			0.47
Low	84.2 (16)	15.7 (3)		36.0 (9)	64.0 (16)	
Medium low	72.7 (8)	27.2 (3)		46.7 (7)	53.3 (8)	
Medium high	77.2 (17)	22.7 (5)		52.4 (11)	47.6 (10)	
High	81.2 (13)	18.7 (3)		29.4 (5)	70.6 (12)	
Adaptation			0.15			0.17
Low	84.6 (22)	15.3 (4)		48.3 (14)	51.7 (15)	
Medium low	68.1 (15)	31.8 (7)		50.0 (12)	50.0 (12)	
Medium high	84.6 (11)	15.3 (2)		29.4 (5)	70.6 (12)	
High	85.7 (6)	14.2 (1)		12.5 (1)	87.5 (7)	
Autonomy			0.0**			0.91
Low	88.8 (16)	11.1 (2)		39.1 (9)	60.9 (14)	
Medium low	73.0 (19)	26.9 (7)		37.9 (11)	62.1 (18)	
Medium high	*87.5 (14)*	12.5 (2)		44.4 (8)	55.6 (10)	
High	*62.5 (5)*	*37.5 (3)*		50.0 (4)	50.0 (4)	
Total classification			0.95			0.35
Low	83.3 (20)	16.6 (4)		43.3 (13)	56.7 (17)	
Medium low	75.0 (12)	25.0 (4)		52.6 (10)	47.4 (9)	
Medium high	80.0 (16)	20.0 (4)		25.0 (5)	75.0 (15)	
High	75.0 (6)	25.0 (2)		44.4 (4)	55.6 (5)	
Access to information about sexuality in school			0.22			0.4
No	85.7 (16)	14.2 (1)		33.3 (7)	66.6 (14)	
Yes	74.5 (38)	25.4 (13)		43.8 (25)	56.1 (32)	
Access to information about sexuality in the family			0.19			0.3
No	77.7 (7)	22.2 (2)		53.8 (7)	46.1 (6)	
Yes	79.6 (47)	20.3 (12)		38.4 (25)	61.5 (40)	

*Significant data. Fischer’s exact test

## Discussion

The present study aimed to evaluate the association between individual and contextual factors and sexual activity in adolescence, also addressing early sexual initiation and inconsistent condom use, behaviors that have been associated with health risks (Dallo & Martins, 2018; Espada et al., 2014). The results showed that adolescents who had sexual debut made more use of alcohol, had repeated a grade at school, belonged to single-parent or extended family configurations and had a greater perception of autonomy in relation to their family. Among those who had not started their sexual life, a perception of higher intrafamily positive affection and a predominance of nuclear families were observed.

Findings indicated the age of the sexual debut did not differentiate vulnerability situations regarding inconsistent condom use. The main difference observed among the group that had already started their sexual life was that early sexual debut was associated to belong to extended family configurations, whereas those who had had sex after the age of 15 were from single-parent families. However, these data should be interpreted with caution, since family compositions cannot be characterized alone as a risk factor for early sexual initiation, given the complexity of the relationships they represent (Costa & Mota, 2012; Oliveira, Siqueira, Dell’Aglio, & Lopes, 2008; Walsh, 2016).

The mean age at the first sexual intercourse was similar to the age found by other Brazilian studies (Dallo & Martins, 2018; Oliveira, Béria, & Schermann, 2014; Silva, Lourdes, Barroso, & Guedes, 2015), as well as by international studies (Espada et al., 2014; Ruiz et al., 2015; Young, Burke, & Gabhainn, 2018). In addition, there were no gender-related differences for any of the variables investigated and the age of sexual debut was similar between boys and girls, contrary to other studies reporting a tendency for boys to initiate sexual activity earlier (Moura et al., 2013; Oliveira et al., 2014; Puente et al., 2011; Silva et al., 2015).

Sexual activity seems to occur in conjunction with other behaviors that may influence adolescent development, such as alcohol/drug use and experimentation, and lower school achievement, as well as family characteristics including its configuration and level of support (Alves, Zappe, & Dell’Aglio, 2015; Bertoni et al., 2009; Dallo & Martins, 2018; Puente et al., 2011; Zappe & Dell’Aglio, 2016). In the present study, the use/experimentation of tobacco and other illicit drugs was also related to the inconsistent condom use, highlighting these behaviors as possible predictors of greater sexual risk.

The last National School Health Survey reported that 19% of the Brazilian adolescents aged 13 to 17 years, among a sample of 10.926, had already tried tobacco and 54.3% had already tried alcohol before the age of 15, with 21% having had at least one episode of drunkenness and 7% having engaged in some conflict with their family due to alcohol use. Other studies have drawn attention to alcohol use among adolescents, warning for the development of dependence (Bertoni et al., 2009; Colder, Shyhalla, & Frndak, 2018; Dallo & Martins, 2018; IBGE, 2016) and the concomitance with other risk behaviors, such as drug use, sexual exposure, and delinquent and self-harm behaviors (Dallo & Martins, 2018; Sasaki, Leles, Matla, Sardinha, & Freire, 2015). However, it is important to point out that more socially vulnerable groups, such as adolescents living in the street or institutionalized adolescents, tend to present higher rates of substance use and earlier sexual debut, indicating that the presence of the family and the school and increased access to health services may be protective factors (Zappe et al., 2018).

In this study, some contextual factors, including family characteristics and socioeconomic and parents’ education levels, were not related to the outcomes investigated. Possible bias could be related to the fact that adolescents were responsible for reporting the socioeconomic and parents’ education levels. However, significant results were found for the dimensions of family support perception.

Affective-consistent families are characterized by a greater ability to cope with problematic situations, verbal or nonverbal expressions of affection, interest in others, and by clear definitions of family rules and members’ roles (Souza et al., 2010). Higher levels in this domain were shown to be related to sexual initiation postponing among the participants. It is plausible to assume that adolescents who perceive these characteristics in their families have more freedom to talk about their affective and sexual concerns and, consequently, choose to wait for a moment of greater security and maturity to initiate their sexual life (Sasaki et al., 2015). Other studies showed that environments with more affective expression, communication, and unity, in which the roles of the members are well defined, with a balance between limits and autonomy, are associated with the lower engagement of the children in sexual risk situations, delinquency, self-harm, and drug use behaviors (Siegel et al., 2014; Souza et al., 2010; Wang et al., 2013).

On the other hand, the autonomy perceived by adolescents was shown to be associated with sexual initiation and early sexual debut in the present study. Autonomy construct concerns the degree of trust, privacy, and freedom among family members (Souza et al., 2010). Accordingly, adolescents who perceive themselves with greater autonomy from the family are those who also see themselves with more freedom to make decisions. However, lower levels of family autonomy could also be seen as protective, since young people still need some degree of parental control and supervision of their actions and experiences (Wang et al., 2013). In addition, grade repetition, which was associated with having had sexual intercourse and early sexual debut, tends to be more common in environments with poor parental supervision, as well as in cases when family relationships are perceived as negative or hostile (Mahendra, Donelli, & Marin, 2018). Difficulties at school and grade repetition have also been commonly indicated as predictors of alcohol and other drug use (Pezzi & Marin, 2017). Therefore, a complex network of associations between these variables is evidenced.

It is known that it is not only greater parental control that will determine lower vulnerability for the children. For example, in a longitudinal study of 913 schoolchildren in New Providence, Bahamas, lower parental monitoring, concerning the parents’ knowledge of their children’s activities, the imposition of rules, and the adolescents’ initiative of reporting their activities to their parents, was a predictor of delinquent behaviors and substance use (Wang et al., 2013). However, parental monitoring was not associated with sexual behavior as communication was more relevant. Therefore, adolescents who perceived they could talk more easily and openly with their parents reported less risky sexual behavior. Poorer communication indicators were also associated with substance use and delinquent behavior, evidencing the importance of this factor for adolescent health (Wang et al., 2013). Taken together, these data suggest that the affective-consistent and family autonomy dimensions may indicate different family dynamics with respect to adolescents’ sexual initiation. Future studies should investigate the existence of different patterns of family support evaluating how they can influence sexual behavior in adolescence.

In addition to the dynamics of the relationships, family configurations were also related to sexual debut in the present study. Belonging to single-parent and extended families was associated with sexual initiation, and the latter with early sexual initiation, corroborating previous findings (Ruiz et al., 2015; Sasaki et al., 2015; Silva et al., 2015). Family configuration alone does not define environments less favorable for child development; however, it cannot be disregarded that, when associated with other social determinants, it may indicate situations of greater vulnerability. In the previous decade, family configurations in Brazil have undergone changes due to the fall in fecundity and the massive presence of women in the labor market (IBGE, 2015). Also, many new family arrangements are still little studied, as is the case of extended families. As a consequence, it would be necessary to know how the existence of other family members, with a degree of kinship or not, could be affecting the sexual education of adolescent children (McGoldrick, Gerson, & Petry, 2012).

Single-parent configurations are, for the most part, characterized by women with their children in Brazil (IBGE, 2015). Common challenges faced by single parents are related to the lack of emotional, social, and material support since most of the time the head of the family needs to manage the tasks related to raising the children, taking care of the house, and being employed. Therefore, these children tend to be autonomous earlier (Anderson, 2016; Verza, 2017). These characteristics may be associated with sexual debut and vulnerability, especially in less economically favorable contexts and where there is more violence (Zappe et al., 2018).

Another important component in the sexual development of individuals is the sexual education received in the family and at school, which was not thoroughly evaluated in this study. Even reporting good levels of access to information about sex and sexuality in both contexts, it was not associated with less engagement in risky sexual behavior among adolescents. Similarly, other studies have shown a lack of relationship between adequate knowledge of preventive practices and attitudes toward HIV/aids and consistent condom use (Brasil, 2013; Moura et al., 2013; Silva et al., 2015), which suggests that information alone is not sufficient to determine healthier behaviors. Thus, other factors, such as those investigated in this study, should be covered by health strategies directed toward adolescents.

The results showed that sexual behaviors in adolescence, although widely investigated in the literature as being a typical and expected characteristic in this age group, are connected not only with their individual characteristics, but are also largely embedded with contextual factors, especially the family and the school. Therefore, the importance of the relationship between the family and the school and of their participation in promoting healthy development for children and adolescents is highlighted. Besides, findings emphasize the urgency of including contextual aspects in sexual education strategies offered by health and education institutions.

## Conclusions

Sexual experience among adolescents is usually associated with risk behaviors by studies of the area and many of these indicate the age of sexual debut as a worrying factor. However, this is not supported by the results found, since the precocity of sexual activity did not distinguish greater exposure to risks. At the same time, our results highlighted that adolescents who had already started sexual activity differed from those who had not, considering both individual (use of alcohol, tobacco, and other drugs and school repetition) and contextual factors (family configuration and autonomy).

The perception of greater autonomy of the adolescent appeared to be linked with early sexual initiation, which indicates that despite the need for families to allow their children to experiment, the supervision and a supportive relationship of affection remain important. Therefore, it is understood that analyzing adolescents’ sexual behavior as a purely individual factor, as if adolescents made their choices in isolation, means overlooking the extensive explanatory potential of the characteristics of the contexts in which they learn about and develop their sexual identity.

Accordingly, it is suggested that the range of the topics covered in sexual education actions goes beyond the merely informative and preventive focus and seeks the promotion of health, covering a diversity of interests and adolescents’ sexual experiences, as well as focusing on sexual pleasure and healthier relationships. Such strategies should also include multiple environments where adolescents live (e.g., families and schools), enabling them to exercise a protective and health promotion role. In addition, the need for greater attention to substance use, especially alcohol use, should be highlighted as those behaviors were frequent among adolescents in this study and in similar ones.

Future studies investigating sexual behaviors during adolescence should consider the inclusion of a broad age range and longitudinal analyses, which would make it possible to evaluate the diversity of trajectories of sexual initiation and their intersection with vulnerability. Also, further research should better investigate the link between context characteristics and the adolescents’ sexual behaviors, evaluating social and family barriers to access information, preventive methods, and health care. Finally, some limitations of the present study should be highlighted. There were a small number of adolescents who had already had sex, which made it impossible to further analyze associations with early sexual initiation and inconsistent condom use outcomes. The fact that the questionnaires were only completed by the adolescents may also be considered a limitation, as sometimes they expressed doubts regarding the socioeconomic status of their families and the education of their parents. This may have generated some bias in these variables, which are commonly related to contexts of greater social vulnerability. Despite this, it is believed that the data revealed the importance of the joint investigation of individual and contextual factors for a better understanding of sexual behaviors in adolescence.

## Data Availability

Data can be requested from the first author by the e-mail address listed in the contact details.

## Abbreviations

IPSF: Inventário de Percepção de Suporte Familiar (Family Perception and Support Inventory)
OR: Odds ratio
PCAP: Pesquisa de Conhecimentos, Atitudes e Práticas (Knowledge, Attitudes and Practices Research)
STIs: Sexually transmitted infections
WHO: World Health Organization

## Footnotes

1 Estimate of monthly household income for the socioeconomic strata. A (R$20,272.56), B1 (R$8695.88), B2 (R$4427.36), C1 (R$2409.01), C2 (R$1446.24), and D (R$639.78).
